# 

**Published:** 1892-02

**Authors:** 


### CONTENTS.

COMMUNICATIONS.

Address by President Betty........ 47
Care of Teeth during Pregnancy and Lactation........ 48
Relationships........ 62
Soliloquy of a Plastidule........ 65
Heredity........ 68
What Causes Variety and Modifications in the Character of Dental Caries........ 86

SELECTIONS.

Lysol........ 91
Impurities in Commercial Samples of Peroxide of Hydrogen........ 92
Bibliographical........93-94
Notice........ 95
Hymeneal........ 95

EDITORIAL.

The Mississippi Valley Dental Association........ 96
A New Disc Mandrel........ 97
The World's Columbian Dental Congress........ 97

